# Review of Canadian species of the genus *Mocyta* Mulsant & Rey (Coleoptera, Staphylinidae, Aleocharinae), with the description of a new species and a new synonymy

**DOI:** 10.3897/zookeys.487.9151

**Published:** 2015-03-16

**Authors:** Jan Klimaszewski, Reginald P. Webster, Caroline Bourdon, Georges Pelletier, Benoit Godin, David W. Langor

**Affiliations:** 1 Natural Resources Canada, Canadian Forest Service, Laurentian Forestry Centre, 1055 du P.E.P.S., P.O. Box 10380, Stn. Sainte-Foy, Quebec, Quebec, Canada G1V 4C7; 2 24 Mill Stream Dr., Charters Settlement, New Brunswick, Canada E3C 1X1; 3 14 A Thomson Rd., Whitehorse, Yukon, Canada Y1A 0C4; 4 Natural Resources Canada, Canadian Forest Service, Northern Forestry Centre, 5320-122 Street, Edmonton, Alberta, Canada T6H 3S5

**Keywords:** Coleoptera, Staphylinidae, Aleocharinae, *Mocyta*, taxonomy, Canada

## Abstract

Six species of the genus *Mocyta* Mulsant & Rey are reported from Canada: *Mocyta amblystegii* (Brundin), *Mocyta breviuscula* (Mäklin), *Mocyta discreta* (Casey), *Mocyta fungi* (Gravenhorst), *Mocyta luteola* (Erichson), and *Mocyta sphagnorum* Klimaszewski & Webster, **sp. n.** New provincial and state records include: *Mocyta breviuscula* – Saskatchewan and Oregon; *Mocyta discreta* – Quebec, Ontario and Saskatchewan; *Mocyta luteola* – New Brunswick, Quebec, Ontario, Massachusetts and Minnesota; and *Mocyta fungi* – Saskatchewan. *Mocyta sphagnorum* is described from eastern Canada from specimens captured in Newfoundland, New Brunswick, Quebec and Ontario. *Mocyta negligens* Mulsant and Rey, a native European species suspected of occurring in Canada, is excluded from the Nearctic fauna based on comparison of European types with similarly coloured Canadian specimens, which are now identified as *Mocyta luteola*. The European species, *Mocyta gilvicollis* (Scheerpeltz), is synonymized with another European nominal species, *Mocyta negligens*, based on examination of type material of the two species. Lectotypes are designated for *Eurypronota discreta* Casey, *Atheta gilvicollis* Scheerpeltz, *Homalota luteola* Erichson, *Colpodota negligens* Mulsant and Rey, *Acrotona prudens* Casey and *Dolosota redundans* Casey. The latter species is here synonymized with *Mocyta luteola*. A review of the six Nearctic species is provided, including keys to species and closely related genera, colour habitus images, images of genitalia, biological information and maps of their distributions in Canada.

## Introduction

There has been considerable confusion about the taxonomic status of the genus *Mocyta* Mulsant & Rey, 1874. Species have historically been assigned to many genera including *Atheta* Thomson, 1858, *Acrotona* Thomson, 1859, *Colpodota* Mulsant & Rey, 1873, *Dolosota* Casey, 1910, *Eurypronota* Casey, 1894, and *Homalota* Mannerheim, 1830. Seevers (1978) included *Mocyta fungi*, and other groups with the pronotal hypomeron strongly deflexed and not visible in lateral view, within the genus *Acrotona*. Casey (1894, 1910) did not formally recognize *Mocyta* as a distinct genus and described several species of *Mocyta* and *Acrotona* in the genera *Eurypronota* Casey and *Dolosota* Casey. Lohse and Smetana (1985) examined types of *Homalota breviuscula* Mäklin from Sitka, Alaska and assigned the species to *Mocyta* as a subgenus of *Atheta*. This species was later recorded as *Mocyta breviuscula* from eastern Canada by Klimaszewski et al. (2005, 2007a, 2008), Webster et al. (2009), and Majka and Klimaszewski (2010). Lohse et al. (1990) recognized *Mocyta* as a distinct genus and reported *Mocyta amblystegii* (Brundin) for the first time from northwestern North America, confirming it as a holarctic species. An adventive Palaearctic species, *Mocyta fungi* (Gravenhorst), is now broadly distributed in Canada and the USA (Muona 1984, Gusarov 2003, McLean et al. 2009, Klimaszewski et al. 2011, 2013).

We believe that species of *Mocyta* constitute a monophyletic evolutionary lineage defined by the shape of the spermatheca, antennal and pronotal structure, and pubescence and punctation patterns. The genus is externally similar to *Acrotona*, *Strigota* and *Atheta*, sharing with the two former genera a strongly deflexed hypomeron on the pronotum, which is not visible in lateral view. Molecular studies by Elven et al. (2012) clearly treat *Mocyta* as a taxon of generic rank within the clade of Athetini. The purpose of this paper is to review all Canadian species of *Mocyta* and to provide modern tools and illustrations for their proper identification. *Mocyta* species are often abundant in forest litter samples and may be used as indicators of forest health.

## Material and methods

Approximately 1000 adults of the genus *Mocyta* from Canada were studied, and most specimens were dissected to examine the genitalic structures that were dehydrated in absolute alcohol, mounted in Canada balsam on celluloid microslides, and pinned with the specimens from which they originated. Images of the entire body and the genital structures were taken using an image processing system (Nikon SMZ 1500 stereoscopic microscope; Nikon Digit-like Camera DXM 1200F, and Adobe Photoshop software).

Morphological terms mainly follow those used by Seevers (1978), Ashe (2000), and Klimaszewski et al. (2011). The ventral side of the median lobe of the aedeagus is considered to be the side of the bulbus containing the foramen mediale, the entrance of the ductus ejaculatorius, and the adjacent ventral side of the tubus of the median lobe with internal sac and its structures (this part is referred to as the parameral side in some recent publications); the opposite side is referred to as the dorsal part. In the species descriptions, microsculpture refers to the surface of the upper forebody (head, pronotum and elytra).

The structure of antennae, body proportions including size, shape and convexity of pronotum, density of punctures on the forebody, and the shape of the spermatheca, particularly that of the capsule with apical invagination, provide the best diagnostic characteristics for species of *Mocyta*. The shape of the median lobe of the aedeagus is generally similar in all species of *Mocyta* occurring in Canada, except for some structures of the internal sac, but several features differ among species, including: the shape of sternite VIII and the form of its basal suture (antecostal suture); the distance between the antecostal suture and the base of the disc; and the shape of the apical part of the disc. In addition, there is great diversity in the form of the spermathecal stem and particularly its posterior part with variable coils and twists within the same species.

### Depository/institutional abbreviations

**AAFC** Agriculture and Agri-Food Canada, Atlantic Cool Climate Crop Research Centre, St. John’s, Newfoundland and Labrador, Canada.

**BGC** Benoit Godin Collection, Whitehorse, Yukon Territory, Canada.

**CNC** Canadian National Collection of Insects, Arachnids and Nematodes, Agriculture and Agri-Food Canada, Ottawa, Ontario, Canada.

**CCL** Centre de Conservation et d’Etude des Collections Musée des Confluences, Lyon, France.

**LFC** Natural Resources Canada, Canadian Forest Service, Laurentian Forestry Centre, R. Martineau Insectarium, Quebec City, Quebec, Canada.

**NHMV** Natural History Museum, Vienna, Austria.

**RWC** Reginald Webster Collection, Charters Settlement, New Brunswick, Canada.

**USNM** United States National Museum, Smithsonian Institution, Washington, D.C., USA.

**ZMB** Zoological Museum of Humboldt University, Berlin, Germany.

**ZML** Museum of Zoology, Lund University, Lund, Sweden.

**ZMH** Zoological Museum Helsinki, Helsinki, Finland.

### Checklist of Canadian *Mocyta* species

New jurisdictional records are indicated in bold type.

**I. *Mocyta amblystegii* species group**

1) *Mocyta amblystegii* (Brundin, 1952), Holarctic. Canada: YT, NT, NU; USA: AK.

2) *Mocyta breviuscula* (Mäklin, 1852), Nearctic. Canada: YT, BC, AB, **SK**, ON, QC, NB, NS, LB, NF; USA: **OR**, AK.

3) *Mocyta fungi* (Gravenhorst, 1806), Palaearctic, adventive in North America, cosmopolitan in many regions of the world. Canada: YT, NU, BC, AB, **SK**, ON, QC, NB, NS, PE, LB, NF; USA: AK.

**II. *Mocyta luteola* species group**

4) *Mocyta luteola* (Erichson, 1839), Nearctic, new Canadian record. Canada: **NB**, **QC**, **ON**; USA: IN, **MA**, **MN**, NY, WI.

**III. *Mocyta discreta* species group**

5) *Mocyta discreta* (Casey, 1894), Nearctic, new Canadian record. Canada: **QC**, **ON**; USA: IA, **MN.**

6) *Mocyta sphagnorum* Klimaszewski & Webster, **sp. n.** Canada: **NF**, **NB**, **QC**, **ON.**

### Distribution

Each species is cited with its currently known distribution in Canada and USA. Data for distribution map (Canada only) were extracted from specimens in collections. Geographic coordinates were standardized using the NAD83 datum, and maps projected onto a Lambert Conic Conformal using ESRI ArcMap version 10 for Windows. The following abbreviations are used in the text for Canadian provinces and territories:

AB – Alberta, BC – British Columbia, LB – Labrador, MB – Manitoba, NB – New Brunswick, NF – Newfoundland (island), NS – Nova Scotia, NT – Northwest Territories, NU – Nunavut, ON – Ontario, PE – Prince Edward Island, QC – Quebec, SK – Saskatchewan, YT – Yukon Territory.

USA state abbreviations follow those of the USA Postal Service.

## Taxonomic review

### Tribe Athetini Casey, 1910

#### Key distinguishing *Mocyta*, *Acrotona* and *Strigota*

[Canadian genera with pronotal hypomeron not visible in lateral view]

**Table:** 

1	Antennae thick, articles V-X more or less transverse (Fig. 1a); body narrowly elongate, densely punctate, particularly on abdomen, dorsal surface with fine white pilose pubescence (Fig. 1a); pronotum approximately as broad as maximum width of elytra (Fig. 1a); tergite VIII in both sexes with the basal line (antecostal suture) joining the base of tergite (Figs 1d, g), and not the sides of the disc as in other aleocharines; apical margin of female sternite VIII with row of strong microsetae on its dorsal side (Fig. 1h)	***Strigota* Casey**
–	Antennae in most specimens thin, articles V-X subquadrate or slightly transverse (Figs 2a–8a); body broadly to narrowly elongate, moderately densely punctate, pubescence different (Figs 2a–8a); pronotum often broader than maximum width of elytra; tergite VIII in both sexes with the basal line joining sides of the disc (Figs 2d, g, 3e, i); apical margin of female sternite VIII with less strongly developed apical microsetae (Figs 3j, 4h)	**2**
2	Antennae very thin and pale, in most specimens contrasting in colour with head, articles V-X subquadrate, transverse to slightly elongate (Figs 3a–8a, e); pronotum broad and shield-shaped, often broader than maximum width of elytra, pubescence moderately dense and directed straight posteriad or obliquely posterolaterad from midline of disc (Figs 3a–8a, e); abdomen gradually narrowed apically and broadly rounded posteriorly; spermatheca with capsule hemispherical or elongate and sac-shaped with usually small apical invagination and short neck, stem thin and regularly or irregularly coiled posteriorly (Figs 3g, h, 4f, 5d–h, 6e–g, 7e, 8f)	***Mocyta* Mulsant & Rey**
–	Antennae normally developed and not appearing very thin, usually not strongly contrasting in colour with head (Fig. 2a); pronotum subquadrate to transverse, approximately as wide as elytra, pubescence usually very dense and directed lateroposteriad from midline of disc (Fig. 2a); abdomen tapering apically and often slightly pointed; spermatheca differently shaped, capsule more or less spherical and extended to elongate neck, stem broader than that in *Mocyta*, regularly coiled posteriorly and often with swelled apex (Fig. 2f)	***Acrotona* Thomson**

**Figures 1a–h.**
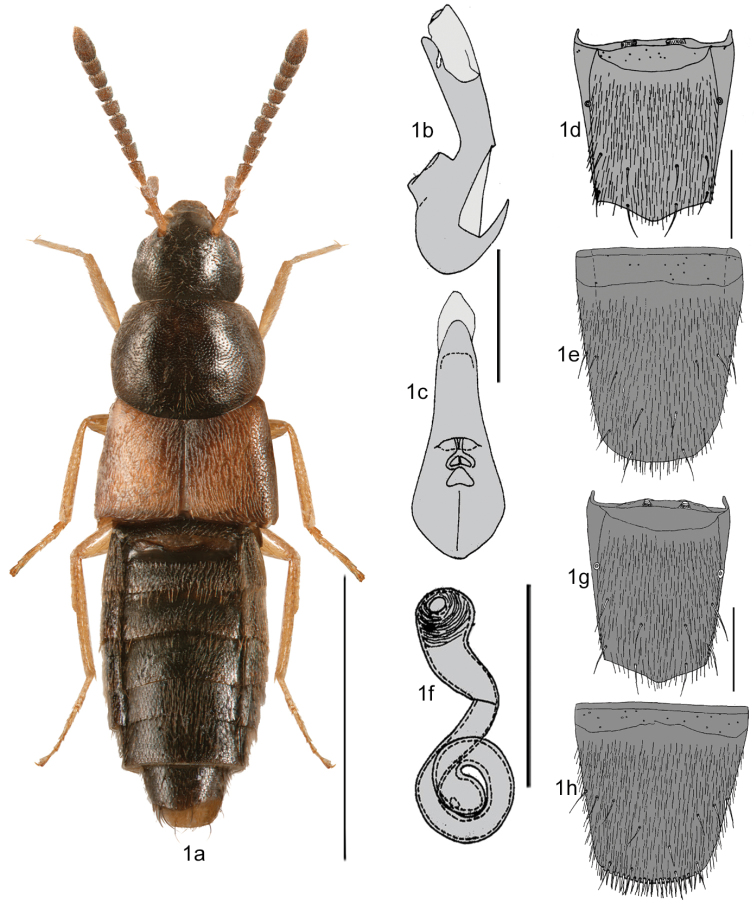
*Strigota ambigua* (Erichson): **1a** habitus in dorsal view **1b** median lobe of aedeagus in lateral view **1c** median lobe of aedeagus in ventral view **1d** male tergite VIII **1e** male sternite VIII **1f** spermatheca **1g** female tergite VIII **1h** female sternite VIII. Figures 1b–h after Gusarov 2003, slightly modified. Scale bar for habitus = 1 mm, and the remaining scale bars = 0.2 mm.

**Figures 2a–h.**
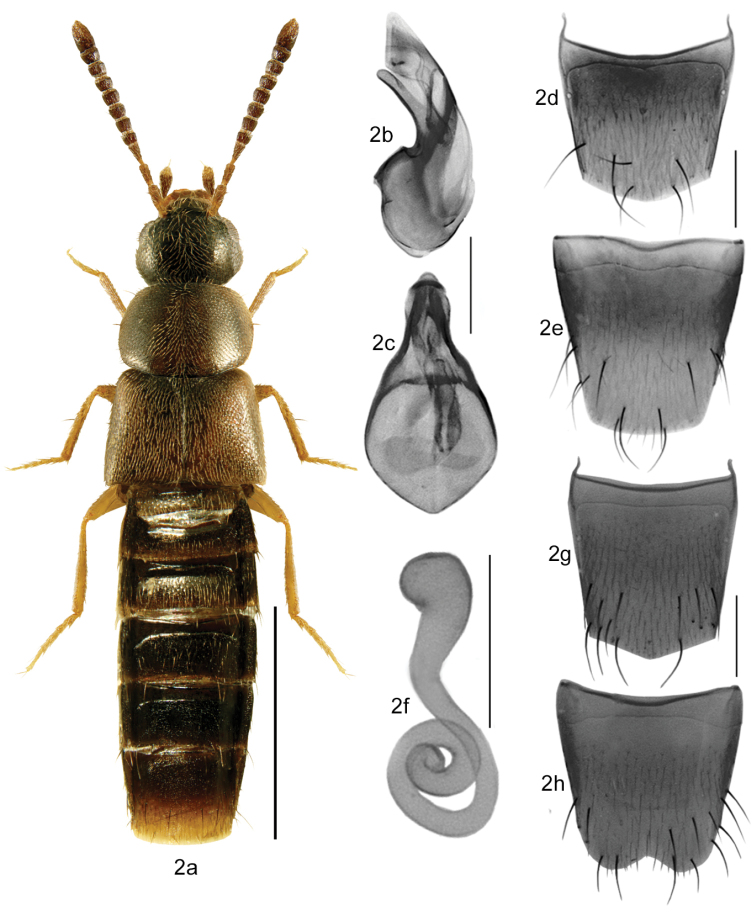
*Acrotona subpygmaea* (Bernhauer): **2a** habitus in dorsal view **2b** median lobe of aedeagus in lateral view **2c** median lobe of aedeagus in dorsal view **2d** male tergite VIII **2e** male sternite VIII **2f** spermatheca **2g** female tergite VIII **2h** female sternite VIII. Scale bar for habitus = 1 mm, and the remaining scale bars = 0.2 mm.

#### Mocyta

Taxon classificationAnimaliaColeopteraStaphylinidae

Mulsant & Rey, 1874

Mocyta For synonymy, see Gusarov 2003, Lohse et al. 1990, Smetana 2004

##### Diagnosis.

**Figures 3a–j.**
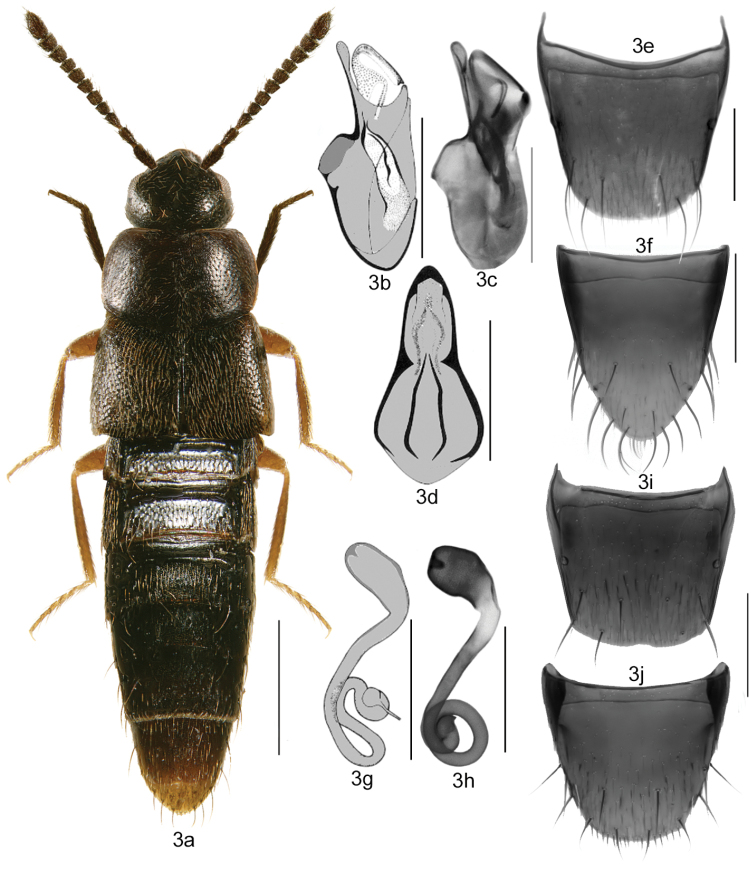
*Mocyta amblystegii* (Casey): **3a** habitus in dorsal view **3b, c** median lobe of aedeagus in lateral view **3d** median lobe of aedeagus in dorsal view **3e** male tergite VIII **3f** male sternite VIII **3g–h** spermatheca **3i** female tergite VIII **3j** female sternite VIII. Scale bar for habitus = 1 mm, and the remaining scale bars = 0.2 mm.

**Figures 4a–h.**
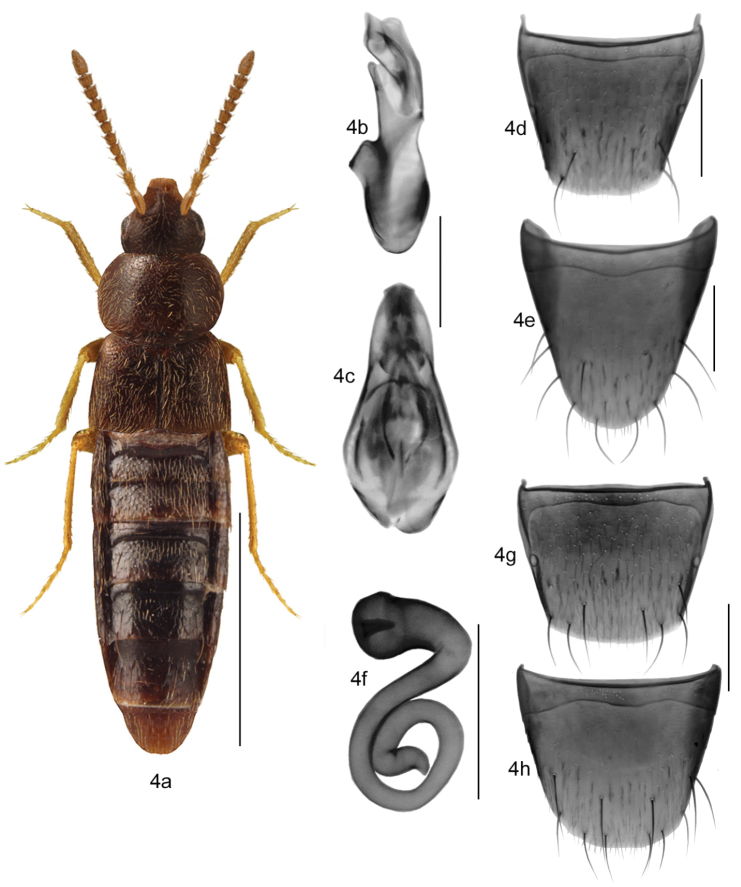
*Mocyta breviuscula* (Brundin): **4a** habitus in dorsal view **4b** median lobe of aedeagus in lateral view **4c** median lobe of aedeagus in dorsal view **4d** male tergite VIII **4e** male sternite VIII **4f** spermatheca in lateral view **4g** female tergite VIII **4h** female sternite VIII. Scale bar for habitus = 1 mm, and the remaining scale bars = 0.2 mm.

**Figures 5a–j.**
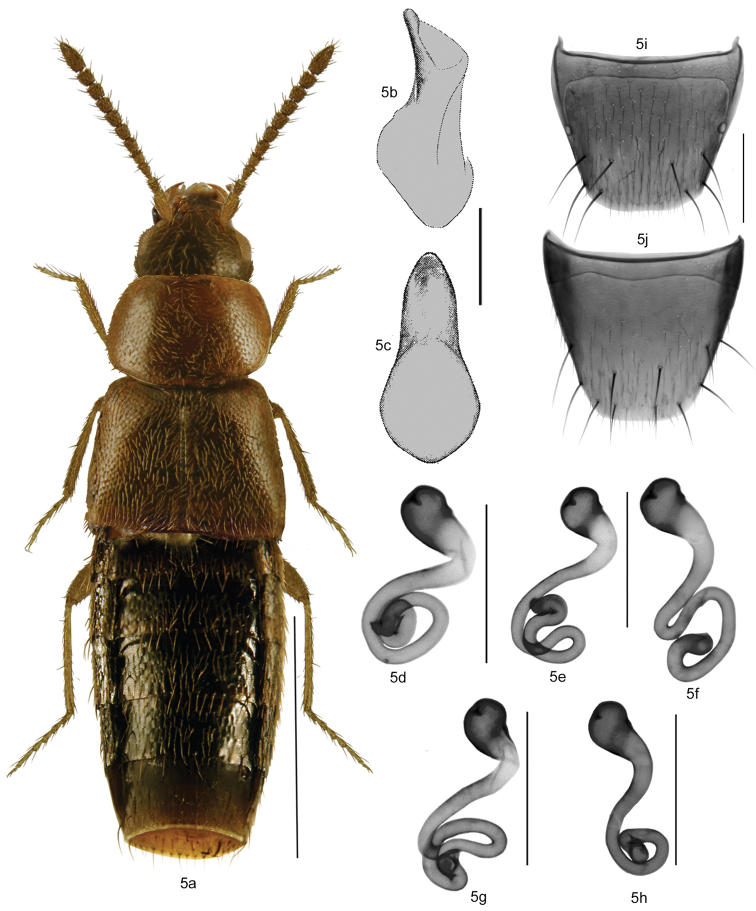
*Mocyta fungi* (Gravenhorst): **5a** habitus in dorsal view **5b** median lobe of aedeagus in lateral view **5c** median lobe of aedeagus in ventral view **5d–h** spermatheca **5i** female tergite VIII **5j** female sternite VIII. Scale bar for habitus = 1 mm, and the remaining scale bars = 0.2 mm.

**Figures 6a–i.**
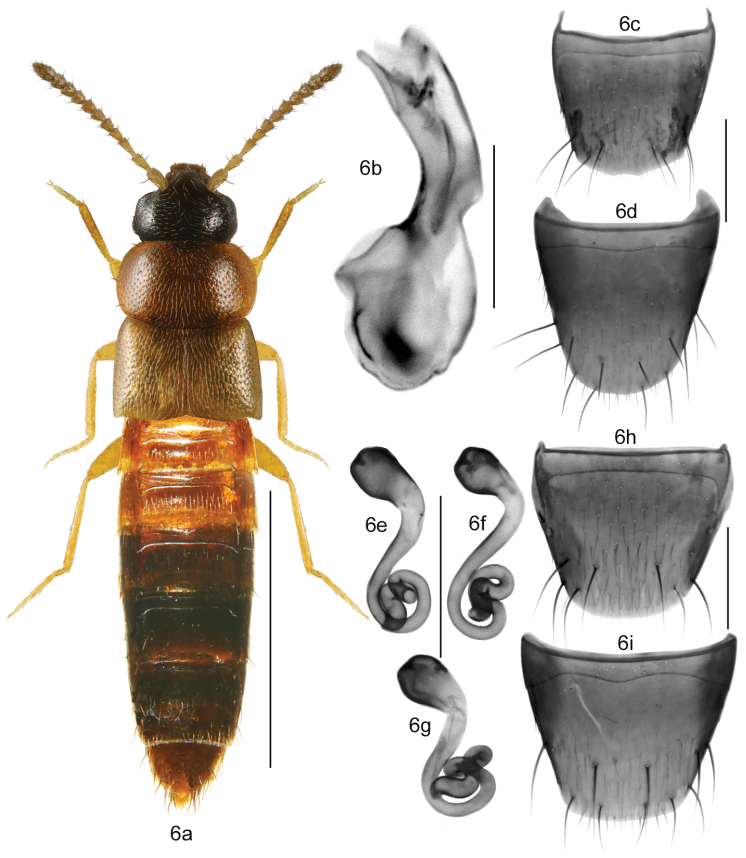
*Mocyta luteola* (Erichson): **6a** habitus in dorsal view **6b** median lobe of aedeagus in lateral view **6c** male tergite VIII **6d** male sternite VIII **6e–g** spermatheca **6h** female tergite VIII **6i** female sternite VIII. Scale bar for habitus = 1 mm, and the remaining scale bars = 0.2 mm.

**Figures 7a–g.**
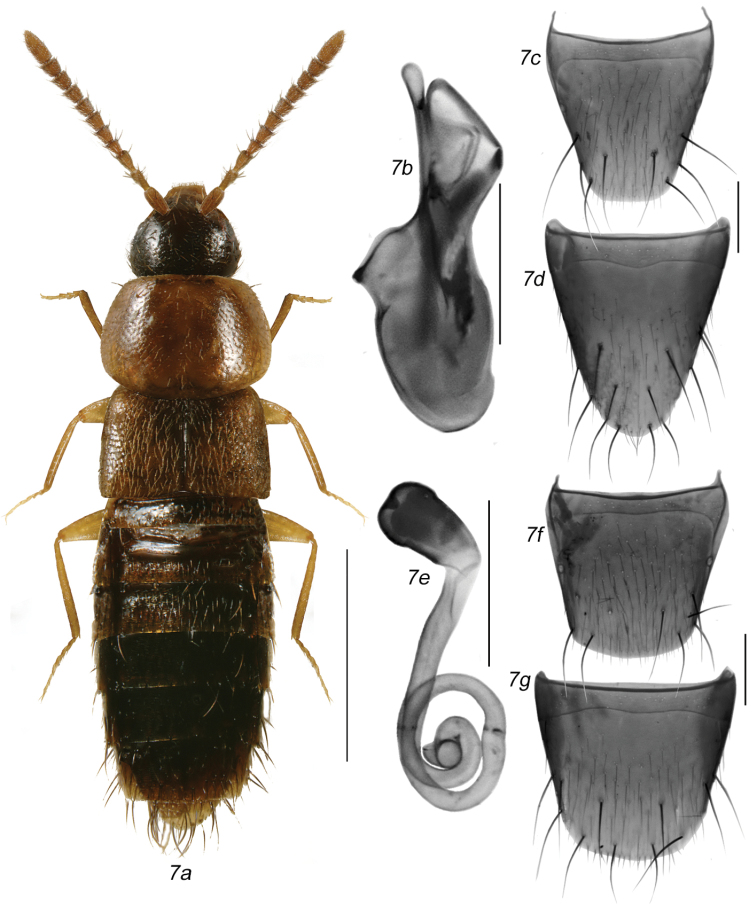
*Mocyta discreta* (Casey): **7a** habitus in dorsal view **7b** median lobe of aedeagus in lateral view **7c** male tergite VIII **7d** male sternite VIII **7e** spermatheca **7f** female tergite VIII **7g** female sternite VIII. Scale bar for habitus = 1 mm, and the remaining scale bars = 0.2 mm.

*Mocyta* may be distinguished from the other genera of Canadian Aleocharinae except for *Acrotona* Thomson and *Strigota* Casey, by having the pronotal hypomeron not visible in lateral view. From *Acrotona* and *Strigota*, as well as other aleocharine genera, it may be distinguished by the following combination of characters: antennae very thin and pale, in most specimens contrasting with body colour (Figs 3a–7a); pronotum glossy, moderately convex, broad and shield-shaped, widest at or near middle, with pubescence directed posteriad in midline or entire central section of disc (Fig. 6a) and posterolaterad at sides, pronotum is at least as broad as the base of the elytra but in most specimens broader (Figs 3a–7a); median lobe of aedeagus of a simple form, tubus *ca.* half length of median lobe, narrowly tapering and rounded apically in dorsal view (Figs 3d, 4c, 5c) and straight and narrow apically in lateral view (Figs 3b, c, 4b, 5b, 6b), internal sac structures inconspicuous, usually elongate and not strongly pronounced (Figs 3b–d, 4b, c, 6b); male tergite VIII truncate apically and without teeth and other secondary sexual characters (Figs 3e, 4d, 5i, 6c), sternite VIII with longer macrosetae than those of females (Figs 3f, 4e, 6d); spermatheca with capsule hemispherical, or elongate and sac-shaped with usually small apical invagination and short neck, stem thin and regularly or irregularly coiled posteriorly (Figs 3g, h, 4f, 5d–h, 6e–g).

The shape of the spermatheca in *Acrotona* is different, with a capsule more or less spherical and extended to a broad and long neck, often pitcher-shaped, and a stem that is broader than that in *Mocyta*, regularly coiled posteriorly and often with a swelled apex (Fig. 2f). *Strigota* may be easily distinguished from *Mocyta* and *Acrotona* by the basal line of the abdominal tergum VIII laterally joining the base of the tergum in both sexes (Figs 1d, g), while in other athetines the basal line is separated from the tergite base (Figs 3e, i). For illustrations, see also Gusarov (2003).

##### Key to Canadian species of the genus *Mocyta*

**Table:** 

1	Body bicoloured, head and at least posterior part of abdomen brown to almost black, and remainder of the body reddish to yellowish-brown, pronotum in most specimens paler than the rest of the body, in some specimens elytra mottled with small and irregular in shape darker spots (Figs 6a, 7a); genital structures as illustrated (Figs 6b, e–g, 7b–e)	**2**
–	Body uniformly brown to black except for paler appendages in most specimens	**3**
2	Pronotum approximately as broad as elytra (Fig. 6a); antennal articles V-X subquadrate (Fig. 6a); median lobe of aedeagus with tubus arcuate and apex pointing ventrally in lateral view (Fig. 6b); spermatheca with capsule sac-shaped and sinuate stem irregularly coiled posteriorly (Figs 6e–g); eastern Canada	***Mocyta luteola* (Erichson)**
–	Pronotum much broader than elytra (Fig. 7a); antennal articles V-X in most specimens slightly elongate; median lobe of aedeagus with tubus straight and apex in horizontal position in lateral view; spermatheca with capsule club-shaped and straight stem coiled posteriorly (Figs 7b, e); eastern Canada	***Mocyta discreta* (Casey)**
3	Elytra distinctly longer than pronotum (Figs 3a, 5a)	4
–	Elytra as long as or slightly shorter than pronotum (Figs 4a, 8a, e)	5
4	Pronotum approximately rectangular in shape, gradually narrowed in apical third of its length (Fig. 5a); spermathecal capsule pear-shaped, rounded apically and gradually narrowed posteriorly, apical invagination small and shallow, stem thin and irregularly twisted posteriorly (Figs 5d–h); only females are present in North America; adventive species broadly distributed across North America and transcontinental in Canada	***Mocyta fungi* (Gravenhorst)**
–	Pronotum approximately trapezoidal in shape, strongly narrowed apically from basal third of its length (Fig. 3a); spermathecal capsule narrowly elongate and sac-shaped, apical invagination small and shallow; stem sinuate and irregularly twisted or coiled posteriorly (Figs 3g, h); median lobe of aedeagus with tubus straight in lateral view (Figs 3b, c); holarctic species known from northwestern Canada and Alaska	***Mocyta amblystegii* (Brundin)**
5	Pronotum as broad as elytra (Fig. 4a); spermatheca with capsule pitcher-shaped and flat apically with elongate apical invagination, stem broadly coiled posteriorly (Fig. 4f); median lobe of aedeagus with tubus straight in lateral view (Fig. 4b); transcontinental in Canada and reported from Alaska, Oregon, California and Nevada	***Mocyta breviuscula* (Mäklin)**
–	Pronotum in many specimens broader than elytra (Fig. 8e); spermatheca with capsule pear-shaped with short apical invagination, stem broadly irregularly coiled posteriorly (Fig. 8f); median lobe of aedeagus with tubus straight and apex in horizontal position in lateral view (Fig. 8b); known from sphagnum in black spruce and cedar forests and swamps; distributed in eastern Canada	***Mocyta sphagnorum* Klimaszewski & Webster, sp. n.**

#### I. *Mocyta amblystegii* species group

**Diagnosis.** Body entirely dark brown to black except for light-coloured appendages; pronotum moderately transverse, approximately as broad as elytra or slightly broader, sides arcuate, pubescence directed posteriad only in midline and obliquely posteriad elsewhere (Figs 3a, 4a, 5a); elytra in most specimens longer than pronotum (Figs 3a, 5a) except for *Mocyta breviuscula* (Fig. 4a); spermatheca and median lobe of aedeagus as illustrated (Figs 3b–d, g, h, 4b, c, f, 5b–h).

##### 1. Mocyta amblystegii

Taxon classificationAnimaliaColeopteraStaphylinidae

(Brundin)

Figs 3a–j

Atheta amblystegii Brundin 1952: 135; Lohse et al. 1990, Smetana 2004.

###### Diagnosis.

Body narrowly oval (Fig. 3a), length 2.5–3.0 mm; uniformly brown to black, appendages light brown (Fig. 3a); antennal articles I-IV elongate and V-X subquadrate or slightly transverse (Fig. 3a); pronotum broad, strongly transverse, rounded laterally and arcuate basally; elytra transverse and at least as long as pronotum; broadly arcuate laterally. MALE: median lobe of aedeagus as illustrated (Figs 3b–d); tergite VIII truncate apically (Fig. 3e); sternite VIII produced apically, with numerous macrosetae and with a broad space between base of disc and antecostal suture, the suture nearly straight or slightly sinuate (Fig. 3f). FEMALE: spermatheca with capsule sac-shaped, as illustrated (Figs 3g, h); tergite and sternite VIII truncate apically (Figs 3i, j).

Adults are externally similar to those of *Mocyta fungi* and may be identified with certainty by the pear-shaped capsule of spermatheca. The presence of males in Canadian populations of *Mocyta amblystegii* and lack of males in Canadian populations of *Mocyta fungi* may also aid in identification of this species.

###### Distribution.

*Mocyta amblystegii* is, according to Lohse (Lohse et al. 1990), a holarctic species recorded in North America from Alaska, Northwest Territories, Yukon and northern Manitoba (Lohse et al. 1990). In Europe, it is recorded from Finland, Norway, and Sweden (Smetana 2004).

###### Natural history.

Adults were found under leaf litter and in moss (Lohse et al. 1990).

##### 2. Mocyta breviuscula

Taxon classificationAnimaliaColeopteraStaphylinidae

(Mäklin)

Figs 4a–h

Homalota breviuscula Mäklin *in* Mannerheim, 1852: 309; Lohse and Smetana 1985: 285, 292 (as Atheta subgenus Mocyta, redescription based on type); Klimaszewski et al. 2011: 107, 218, 246; Gusarov 2003: 100–102. **LECTOTYPE** (male): UNITED STATES, Alaska, Sitka (Sitcha); Lectotype, Lohse designation 1983 (ZMH). An extensive list of synonymies for *Mocyta breviuscula* is provided by Gusarov 2003: 101.
Acrotona prudens Casey 1910: 149; synonymized by Lohse and Smetana 1985: 293. Type localities: British Columbia, Queen Charlotte Islands and Metlakatla (Casey 1910: 149). **LECTOTYPE** (female): 2 CI [Queen Charlotte Islands], Type USNM 38985 (USNM), present designation.

###### Diagnosis.

Body narrowly oval (Fig. 4a), length 2.4–3.0 mm; body uniformly dark brown to almost black and often with reddish tinge, appendages yellowish to reddish-brown; antennal articles I-IV elongate and V-X subquadrate; pronotum transverse, arcuate laterally and arcuate basally; elytra transverse and nearly as long as pronotum; abdomen broadly arcuate laterally. MALE: Median lobe of aedeagus as illustrated (Figs 4b, c); tergite VIII truncate apically (Fig. 4d); sternite VIII slightly produced apically with broad space between base of the disc and antecostal suture, the suture more or less sinuate (Fig. 4e). FEMALE: spermatheca with capsule pitcher-shaped and flat apically with elongate apical invagination, stem broadly coiled posteriorly (Fig. 4f); tergite and sternite VIII truncate apically (Figs 4g, h).

The combination of uniform body colour, elytra no longer than pronotum, distinct shape of spermatheca with deep capsular invagination, and shape of male sternite VIII with broad space between base of disc and antecostal suture, can distinguish *Mocyta breviuscula* from the remaining Nearctic congeners.

###### Distribution.

*Mocyta breviuscula* is a native Canadian species distributed transcontinentally in northern Canada, and it was also reported from Alaska, California and Nevada (Lohse and Smetana 1985, Lohse et al. 1990, Gusarov 2003, Webster et al. 2009, Klimaszewski et al. 2005, 2007b, 2008, 2011, Majka and Klimaszewski 2008, Brunke et al. 2012). We include new records of this species from Saskatchewan and Oregon (see below for new distribution localities).

###### Natural history.

In Newfoundland, adults were frequently caught in pitfall traps in various forest types (birch, spruce-lichen, spruce-poplar, fir), in vegetation on coastal sand dunes, on shrubby limestone barrens and in disturbed fields amongst grass and weeds (Klimaszewski et al. 2011). The activity period is June to September. Adults were captured in pitfall traps from June to August in yellow birch/balsam fir forest in southern Quebec and in sphagnun and litter in an eastern white cedar swamp in New Brunswick (Klimaszewski et al. 2005, 2007b, Webster et al. 2009).

###### New jurisdictional records.

CANADA: Saskatchewan, Saskatoon, 28.IX.1976, E.J. Kiteley (CNC) 1 male.

UNITED STATES: Oregon, Grant Co., Strawberry Range, Strawberry Lake, 1920 m, 1.VI.1989, A. Smetana, NA21(CNC)3 males, 1 female.

##### 3. Mocyta fungi

Taxon classificationAnimaliaColeopteraStaphylinidae

(Gravenhorst)

Figs 5a–j

Aleochara fungi Gravenhorst 1806: 157; Muona 1984, Gusarov 2003, Smetana 2004, McLean et al. 2009, Klimaszewski et al. 2011. For extensive synonymy, see Gusarov 2003 and Smetana 2004. **LECTOTYPE** (female): *Aleochara fungi* Gravenhorst; Lectotype, V. Mahler des. 1986; Europa, nr. 5499; typus; *fungi* Gr. (ZMB) [examined by Klimaszewski].

###### Diagnosis.

Body broadly oval (Fig. 5a), length 2.4–3.0 mm; body uniformly dark brown to black, in some specimens body black and posterior or central part of elytra with reddish tinge, appendages light brown; antennal articles I-IV elongate and V-X subquadrate or slightly transverse; pronotum broad, transverse, rounded laterally and arcuate basally; elytra transverse and *ca.* as long as pronotum or longer; abdomen broadly arcuate laterally (Fig. 5a). MALE: median lobe of aedeagus as illustrated (Figs 5b, c) [absent in North America]. FEMALE: spermatheca with capsule pear-shaped, as illustrated (Figs 5d–h); tergite VIII truncate apically (Fig. 5i); sternite VII broadly rounded apically with fringe of microsetae, distance between antecostal suture and base of disc narrow, antecostal suture sinuate (Fig. 5j).

This species is externally very similar to *Mocyta amblystegii* and may be identified with certainty only by the shape of the spermatheca. The presence of males in Canadian populations of *Mocyta amblystegii* and lack of males in Canadian populations of *Mocyta fungi* may also aid in the identification of these species.

###### Distribution.

Palaearctic, adventive in North America, cosmopolitan in many regions of the world (Smetana 2004). Canada: YT, NU, BC, AB, **SK**, ON, QC, NB, NS, PE, LB, NF, and USA: AK, ME, MA, MN, NY, OR, RI (Moore and Legner 1975, Muona 1984, Gusarov 2003, Klimaszewski et al. 2005, 2007a, 2008, 2011, 2012, Majka and Klimaszewski 2008, 2010, Brunke et al. 2012). We include new records of this species from Saskatchewan in Canada.

###### Natural history.

*Mocyta fungi* is represented in North America by parthenogenetic females only. In Newfoundland, adults were collected in pitfall traps in cut and burned balsam fir, birch, spruce-poplar and riparian forests, in agricultural fields and amongst vegetation on coastal sand dunes (Klimaszewski et al. 2011). The adult activity period in Newfoundland is June to September. Adults were captured by pitfall traps from May to September in forest litter in mixed wood, red spruce in New Brunswick and yellow birch forest in southern Quebec (Klimaszewski et al. 2005, Majka and Klimaszewski 2010).

###### New jurisdictional records.

CANADA: **Saskatchewan:** Cypress Hills, wet willow stand, 49,5978°, -109,9231°, 1134 m, 2.IX.2012, 2 males; wet pond, riparian, 49,6704°, -109,5005°, 1189 m (LFC) 1 female.

#### II. *Mocyta luteola* species group

**Diagnosis.** Pronotum strongly transverse, 1.5 times broader than long, sides arcuate, pubescence directed posteriad in midline and central part of the disc (Fig. 6a); elytra approximately as wide as pronotum (Fig. 6a); spermatheca and median lobe of aedeagus as illustrated (Figs 6b, e–g).

##### 4. Mocyta luteola

Taxon classificationAnimaliaColeopteraStaphylinidae

(Erichson)

Figs 6a–i , Map 1

Homalota luteola Erichson 1839: 114 [habitat in America septentrionalis, Dom. Zimmerman]; Bland 1865: 397; Blatchley 1910: 353; Moore and Legner 1975: 365. **LECTOTYPE** (male): USA: Am.[America] spt.[septentrionalis], Zimm. [Zimmerman]; #5432; Zool. Mus. Berlin.; typus; Lectotype male *Homalota luteola* Erichson, V.I. Gusarov des. 2003 [designation not published]; our lectotype designation label (ZMB) present designation. **PARALECTOTYPES:** labelled as lectotype (ZMB) 1 male, 1 female, present designation.
Dolosota redundans Casey 1910: 137; Moore and Legner 1975: 372. **syn. n. LECTOTYPE** (female): United States of America: NY [New York]; *redundans* Casey; Type USNM 39197; Casey bequest 1925; *Dolosota redundans* Casey - Lectotypus des. Gusarov 2000. *Acrotona luteola* (Er.) V.I. Gusarov det. 2000. We have added J. Klimaszewski’s lectotype, present designation label because Gusarov’s designation was never published (USNM). **PARALECTOTYPES:** United States of America: NY [New York]; *redundans* Casey; Type USNM 39197(USNM)2 females, present designation.

###### Diagnosis.

Body narrowly elongate (Fig. 6a), length 1.8–2.6 mm; head and posterior part of abdomen from brown to almost black, pronotum and basal half of abdomen light yellowish-brown to reddish brown, elytra yellowish to reddish-brown with some irregular small dark brown spots; legs and palps yellowish-brown and antennae either uniformly yellowish or basal articles I-IV yellowish and apical ones light brown; antennal articles I-IV elongate and V-X subquadrate to slightly transverse; pronotum short, transverse, strongly rounded laterally, and arcuate basally (Fig. 6a); elytra *ca.* as long as pronotum (Fig. 6a); abdomen broadly arcuate laterally. MALE: median lobe of aedeagus as illustrated (Fig. 6b); tergite VIII truncate apically, distance between base of disc and antecostal suture moderate in width, suture slightly sinuate medially (Fig. 6c); sternite VIII rounded apically (Fig. 6d). FEMALE: spermatheca with capsule small, pear-shaped and with shallow invagination, stem thin and twisted posteriorly, twists are irregular in shape or forming more or less regular coils (Figs 6e–g); tergite VIII truncate apically (Fig. 6h); sternite VIII broadly rounded apically with apical fringe of short microsetae, distance between base of disc and antecostal suture narrow, suture strongly sinuate medially (Fig. 6i).

###### Distribution.

**Maps 1–3.**
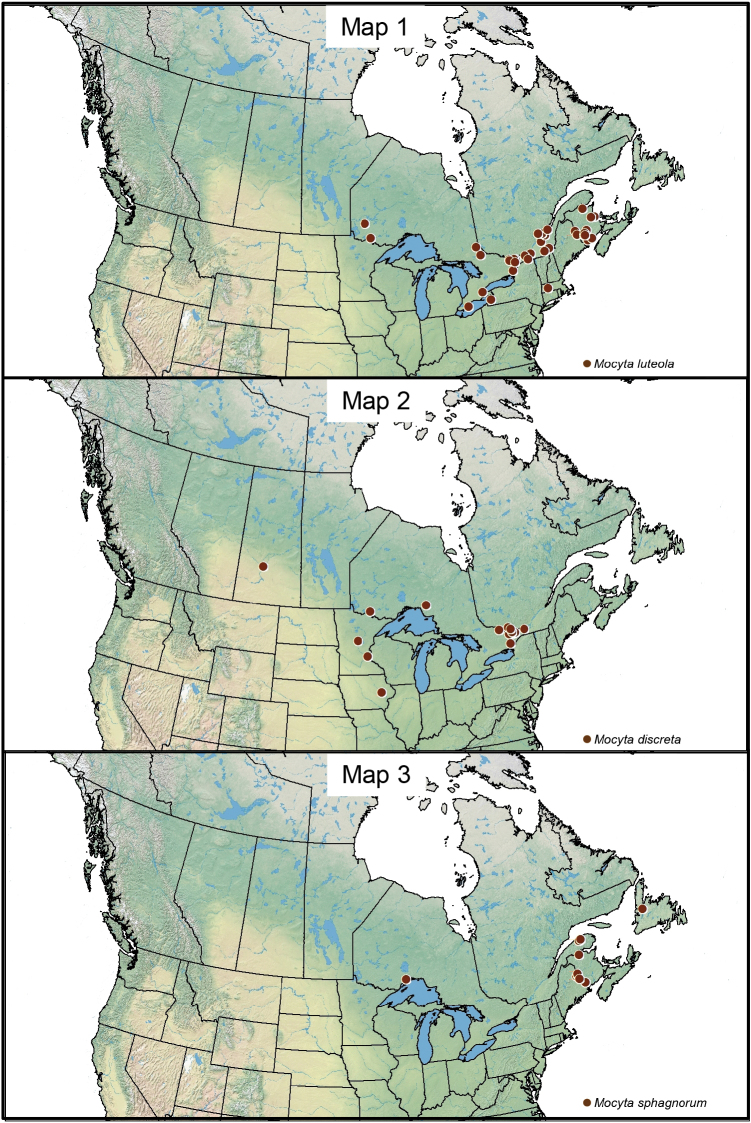
**1** Distribution of *Mocyta cupiens* (Casey) in Canada **2** Distribution of *Mocyta discreta* in Canada **3** Distribution of *Mocyta sphagnorum* in Canada.

This native Nearctic species is reported in Canada for the first time from New Brunswick, Quebec, and Ontario (Map 1). In the USA, new records are provided for Massachusetts and Minnesota, and an additional record is provided for New York. The species was previously reported from Indiana, Michigan, New York and Wisconsin (Erichson 1839, Casey 1910, Bland 1865, Blatchley 1910, Moore and Legner 1975).

###### Natural history.

Most adults from Quebec were collected in yellow birch and balsam fir dominated forest using pitfall traps (Klimaszewski et al. 2007b). In New Brunswick, adults were found: under decaying seaweed on sea beach; under driftwood on a riverbank; in grass, moss and leaf litter near water in alder and cedar swamps and *Carex* marshes; in *Spagnum* moss and leaf litter in a young regenerating mixedwood forest; and in other decaying material in forests. In Ontario, adults were captured in litter around raspberry bushes near a bog, in a *Typha* marsh, and in a nest of *Microtus pennsylvanicus*. Adults were active from March to October in Canada. In Minnesota, adults were captured on a lakeshore and in a *Microtus* nest, and in Indiana were taken by sifting dump vegetable debris from March to November (Blatchley 1910).

###### Locality data.

CANADA: **New Brunswick:** Carleton Co., Bell Forest, 46.2152°N, 67.7192°W, 11.V.2005, river margin, under drift material, M.-A. Giguère & R. Webster (RWC) 1 sex undetermined; Charlotte Co., ca. 9 km NW of New River, 45.2096°N, 66.6483°W, 13.VI.2005, alder swamp near large brook, in grass and leaf litter, R.P. Webster (RWC) 1 male. Kent Co., Kouchibouguac Nat. Pk., 21.IX.1977, D.B. Lyons (CNC) 1 female; same data except A. Smetana (CNC) 1 female, and S.J. Miller (CNC) 1 female. Northumberland Co., Goodfellow Brook P.N.A., 46.8943°N, 65.3796°W, 23.V.2007, old-growth eastern white cedar swamp, in litter & grasses & moss on hummocks near water, R.P. Webster (RWC) 1 female; Restigouche Co., Jacquet River Gorge P.N.A., 47.8200°N, 66.0015°W, 13.V.2010, *Carex* marsh, under alders in leaf litter & moss near brook, R.P. Webster (RWC) 1 female; Saint John Co., Taylor’s Island, 45.2238°N, 66.1265°W, 26.VII.2004, sea beach, under decaying seaweed, R.P. Webster (RWC) 1 sex undetermined; Sunbury Co., 46.0173°N, 66.3741°W, 18.VI.2007, Road 16 regenerating forest, 8.5 year-old regenerating mixed forest, in sphagnum & leaf litter, R.P. Webster (RWC) 1 female; York Co., Canterbury, trail to Browns Mtn. Fen, 5.8978°N, 67.6273°W, 2.V.2005, Mature cedar forest near stream, sifting leaf litter, M. Giguère (RWC) 1 female; Rt. 645 at Beaver Brook, 45.6860°N, 66.8668°W, 3.V.2008, *Carex* marsh in litter at base of dead red maple, R.P. Webster (RWC) 1 female; Charters Settlement, 45.8395°N, 66.7391°W, 14.VI.2008, mixed forest, in decaying (moldy) corncobs & cornhusks, R.P. Webster (RWC) 1 female; 8.5 km W of Tracy, off Rt. 645, 45.6821°N, 66.7894°W, 6.V.2008, wet alder swamp, in leaf litter & grass on hummocks, R.P. Webster (RWC) 1 female. **Quebec:** Blandford, 13.V.1971, E.J. Kiteley (CNC) 1 female; Hudson Heights, 24–30.VII.1956, Lindberg (CNC) 1 female; Montreal, 22.IX.1968, E.J. Kiteley (CNC) 1 male; Montreal, 30.IX.1968, E.J. Kiteley (CNC) 8 females; Montreal, 16.V.1969, E.J. Kiteley (CNC) 2 females; Montreal, 5.X.1979, E.J. Kiteley (CNC) 1 female; Montreal, 4.V.1980, E.J. Kiteley (CNC) 1 female; Ormstown, 22.VIII.1980, E.J. Kiteley (CNC) 1 female; Rigaud end Ch. de la Croix. 5.V.1988, A. & Z. Smetana (CNC) 2 females; Saint-Etienne, Lévis, 6.VI.1981, C. Chantal (CNC) 1 female; Ste-Catherine, Port., 5.VIII.1961, J.C. Aubé (CNC) 4 females; Scotstown, 29.V.2006, C. Levesque (LFC) 4 females, 1 sex?; Mcy Co., St-Joachim, 11.VI.1963, C. Chantal (CNC) 1 female; Sherbrooke, 20.IX.1972, Dondale and Redner (CNC) 1 female; Portneuf, ZEC Batiscan-Nelson, SSAM project, Sapinière à bouleau jaune, Lac des Étangs, 4 gaps, Pitfall trap 21, 46°58'08"N, 72°02'57"W, 30.VI–07.VII.2008, 1 specimen; Pitfall trap 23, 46°58'08"N, 72°02'57"W, 1 specimen; Lac Poissonneux, clear cut, Pitfall trap 66, 47°02'48"N, 72°07'29"W, 297 m, 12.VIII–19.VIII.2008, 1 specimen; 2 gaps, Pitfall trap 69, 47°02'N, 72°07'W, 15.VII–22.VII.2008, 1 specimen; Clear cut, Pitfall trap 95, 47°02'N, 72°07'W, 1 specimen; 2 gaps, Pitfall trap 98, 47°02'N, 72°07'W, 22.VII–29.VII.2008,1 specimen; 29.VII–05.VIII.2008, 1 specimen; 25.VI–02.VII.2008, 2 specimens; Pitfall trap 99, 27.V–03.VI.2008, 1 specimen; Pitfall trap 100, 12.VIII–19.VIII.2008, 1 specimen; Pitfall trap 101, 02.VII-08.VII.2008, 1 specimen; Pitfall trap 102,12.VIII–19.VIII.2008, 1 specimen; 22.VII-29.VII.2008, 1 specimen; 8 gaps, Pitfall trap 103, 47°02'N, 72°07'W, 22.VII-29.VII.2008, 1 specimen; Pitfall trap 105, 25.VI–02.VII.2008, 1 specimen; Pitfall trap 107, 25.VI-02.VII.2008, 1 specimen; 4 gaps, Pitfall trap 109, 47°02'N, 72°07'W, 25.VI–02.VII.2008, 2 specimens; 08.VII–15.VII.2008, 1 specimen; Pitfall trap 110, 1 specimen; 10.VI-17.VI.2008, 2 specimens; Pitfall trap 111, 17.VI–25.VI.2008, 1 specimen; 25.VI-02.VII.2008, 1 specimen; Pitfall trap 113, 08.VII–15.VII.2008, 1 specimen; 10.VI-17.VI.2008, 1 specimen.

**Ontario:** Ancaster, 28.III.1963, J.E.H. Martin (CNC) 4 females; Carleton Co., Fitzroy Prov. Pk., 2-3.V.1979, A. & Z. Smetana (CNC) 1 female; Mer Bleue, 3.III.1973, Redner and Starr (CNC) 27 females; Ottawa, Mer Bleue bog, 16.IV.1972, litter around raspberry, L. LeSage (CNC) 4 females, 1 sex?; Mer Bleue, 17.X.1980, en fauchant herbages dans un champ, L. LeSage (CNC) 7 females; Ottawa, 22.VIII.1912, Beaulieu (CNC) 1 female; Ottawa, Shirleys Bay, 2.V.1970, A. & Z. Smetana (CNC) 1 female; Ottawa, Mer Bleue bog, 23.IV.1982, ridge litter, L. LeSage (CNC) 5 females; Carlsbad Springs, Mer Bleue, 23.V.1980, A. Smetana (CNC) 9 females; Kinburn, 8.VI.1962, ex *Microtus* nest, J.E.H Martin (CNC) 9 females; Ottawa, Black Rapids, 19.VIII.1959, J.R. Vockeroth (CNC) 3 females; Osgoode, 20.X.1967, ex nest of *Microtus pennsylvanicus*, J.M. Campbell and A. Smetana (CNC) 7 females; 6 mi. W. Richmont, 28.III.1973, J.E.H. Martin (CNC) 1 female; Rondo Provincial Park, Marsh Trail, 2.VI.1985, tread *Typha* in marsh, A. Davies and J.M. Campbell (CNC) 1 female; South March, 19.X.1967, A. Smetana (CNC) 1 female; 19 mi. S. Temagami, 1-13.VIII.1973, J. Redner and C. Starr (CNC) 1 female; North Bay, 11.VII.1972, E.J. Kiteley (CNC) 1 female.

UNITED STATES OF AMERICA: **Massachusetts:** Northampton, 5.XI.1978, E.J. Kiteley (CNC) 2 females; **Minnesota**, Minneapolis, 25.VI.1958, E.J. Kiteley (CNC) 1 female; New York: Chautauqua Co., Lake Shore, Sheridan, II.1968, ex nest of *Microtus pennsylvanicus*, A.H. Benton (CNC) 1 female.

###### Comments.

In new material of *Mocyta* from Quebec and New Brunswick, we discovered an unrecorded bicoloured species from Canada that was similar in body size, coloration and shape of spermatheca to the native *Mocyta luteola* (Erichson) and the European *Mocyta negligens* (Mulsant & Rey) and *Mocyta gilvicollis* (Scheerpeltz). After examining the types and additional specimens of the two European species and *Mocyta luteola* and comparing them with Canadian individuals of our new species, we have concluded that our populations represent *Mocyta luteola* and that they are not conspecific with the two European species, as they differ in external morphological features such as body proportions, microsculpture, and shape and pubescence of pronotum. After examining the types of both nominal species (*Mocyta negligens*, *Mocyta gilvicollis*), and additional specimens from Europe, we found no significant morphological differences between the two species. Therefore these two European species are considered as conspecific, and *Mocyta gilvicollis* is considered as a new synonym of *Mocyta negligens* with details listed below (Figs 9a–g, 10–14).

*Colpodota negligens* Mulsant and Rey 1873: 156 (Figs 10–14); Benick and Lohse 1974 (as *Mocyta*); Smetana 2004 (as *Acrotona*).

LECTOTYPE (male): the specimen does not have any original label but it is from the historical Rey collection (CCL) and it is pinned next to the original name label by Rey. It bears V. Gusarov’s lectotype designation label (2000), and his identification label as *Atheta fungi* (Gravenhorst), 2000. Because this designation was never published, we formally designate this specimen as a lectotype and put our determination label as *Mocyta negligens* (Mulsant and Rey), J. Klimaszewski 2014.

PARALECTOTYPES: there are 4 syntypes (1 male, 3 females) in Rey’s collection that are here designated as paralectotypes. One of the syntypes (female) bears a black dot label, which indicates that the specimen was taken in Provence, in southeast France. The specimens bear Paralectotype designation labels by V. Gusarov (2000) but because these designations were not published, we formally designate them as paralectotypes. All are determined as *Mocyta negligens* (Mulsant and Rey), det. J. Klimaszewski 2014.

*Atheta gilvicollis* Scheerpeltz 1949: 355 (Figs 9a–g). **syn. n.**

LECTOTYPE (male): Typus; Atheta (Acrotona) gilvicollis; O. Scheerpeltz [red label]; female sign; Üttligen; IX.1943; ex coll. Scheerpeltz [blue card] (MNHV) examined, present designation.

PARALECTOTYPES: Erlach; X.1951; male sign; *gilvicollis* ex coll. Scheerpeltz [blue card]; Vienna Museum (NHMV) 1 male, examined; Frauenfeld; VII.1955; *gilvicollis* Scheerpeltz, ex coll. Scheerpeltz (NHMV) sex undetermined, examined, present designation.

#### III. *Mocyta discreta* species group

**Figures 8a–h.**
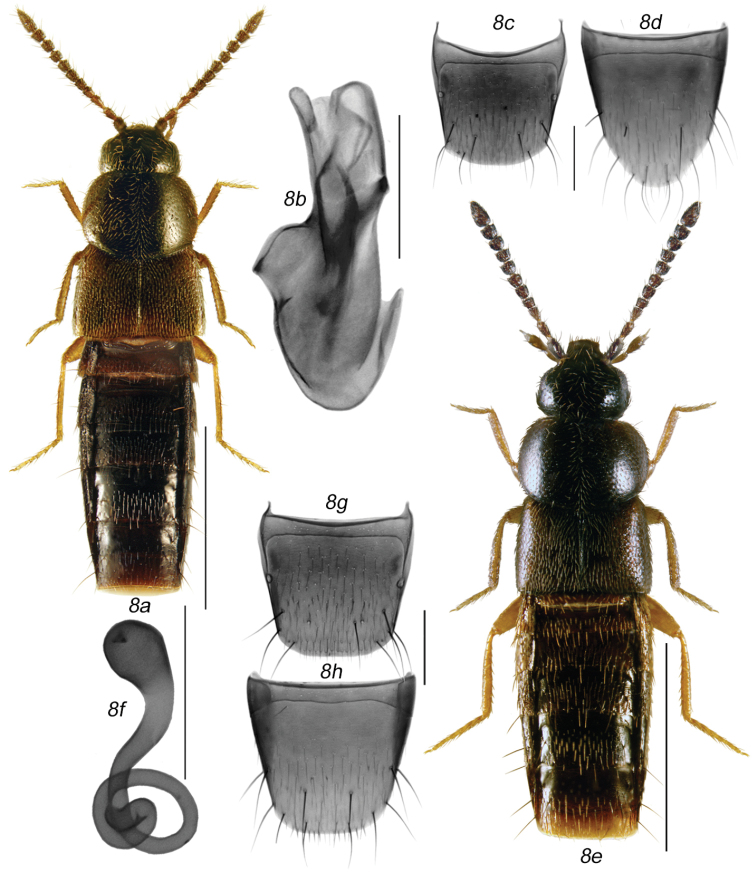
*Mocyta sphagnorum* sp. n.: **8a** male habitus in dorsal view **8b** median lobe of aedeagus in lateral view **8c** male tergite VIII **8d** male sternite VIII **8e** female habitus in dorsal view **8f** spermatheca **8g** female tergite VIII **8h** female sternite VIII. Scale bar for habitus = 1 mm, and the remaining scale bars = 0.2 mm.

**Diagnosis.** Pronotum large, transversely orbicular with rounded lateral and hind angles, usually much broader than elytra, pubescence directed posteriad only in midline and obliquely posteriad elsewhere (Figs 7a, 8a, e); elytra short, as long as or shorter than pronotum (Fig. 7a); median lobe of aedeagus and spermatheca as illustrated (Figs 7b, e, 8b, f).

##### 5. Mocyta discreta

Taxon classificationAnimaliaColeopteraStaphylinidae

(Casey)

Figs 7a–g , Map 2

Eurypronota discreta Casey 1894 [1893]: 335; Moore and Legner 1975: 359 (as *Acrotona*). **LECTOTYPE** (male): USA: Ia [Iowa], Cedar Rapids, Dr. E. Brendel [in orig. description]; *Eurypronota discreta* Casey; Casey bequest 1925; Lectotype label designated by V.I. Gusarov, 1999, but because he never published his designation, we here formally designate this specimen as a lectotype with J. Klimaszewski’s designation label 2014 (USNM). **PARALECTOTYPES:** USA: Ia [Iowa], paratype 2(USNM)1 female; Ia, paratype 3(USNM)1 female; Ia, paratype 4(USNM)1 female; Ia, paratype 5(USNM)1 male; Ia, paratype 6(USNM)1 female; Ia, paratype 7(USNM)1 female; and Ia, paratype 8(USNM)1 female. All these specimens bear V.I Gusarov paralectotype labels, but because he never published his designations we here formally designate these specimens as paralectotypes with J. Klimaszewski’s designation label 2014 (USNM).

###### Diagnosis.

Body broadly oval (Fig. 7a), length 2.4–2.8 mm; head and entire abdomen or its basal part only from brown to almost black, pronotum and basal half of abdomen in most specimens light brown, testaceous or reddish-brown, elytra yellowish to reddish-brown with some irregular small dark brown spots and darker than pronotum, legs and palps yellowish to reddish-brown and antennae either uniformly yellowish to light brown; antennal articles I-IV elongate and V-X variable in length from subquadrate to slightly elongate (Fig. 7a); pronotum transverse, usually very large but variable in width, from slightly broader than elytra to 1/7 wider [pronotum usually broader in females than in males], strongly rounded laterally, and arcuate basally; elytra transverse and shorter than pronotum; abdomen broadly arcuate laterally and with very strong macrosetae apically. MALE: median lobe of aedeagus as illustrated (Fig. 7b); tergite VIII truncate apically (Fig. 7c); sternite VIII slightly produced and rounded apically and with numerous strong macrosetae in apical part of disc, space between base of disc and antecostal suture broad, antecostal suture sinuate medially (Fig. 7d). FEMALE: spermatheca pear-shaped with small and shallow apical invagination, stem thin and straight anteriorly and coiled posteriorly (Fig. 7e); tergite and sternite VIII truncate apically (Fig. 7f, g).

This species is readily recognisable from other *Mocyta* species by its bicoloured body, large pronotum, very strong macrosetae on the apical part of the abdomen, and antennal articles V-X subquadrate to elongate.

###### Distribution.

This nearctic species is newly reported from Canada and the provinces of Ontario, Quebec and Saskatchewan (Map 2), and from Minnesota. Casey (1894) described this species from Cedar Rapids, Iowa, USA, and no other records of this species were published from North America until now.

###### Natural history.

In Ontario, adults were collected in forest litter, deciduous leaf mold, and maple forest from March through October. In Quebec, adults were found in maple-oak forest litter and other deciduous tree litter, from May through August. In Saskatchewan, adults were collected from deciduous forest litter in October.

###### New jurisdictional records.

CANADA: **Ontario:** 7 km W Petawawa, 16.IV.1988, A. Smetana (CNC) 1 male; Chaffeys Locks, 24.X.1971, forest litter, S. Peck (CNC) 4 females, 1 male, 2 sex undetermined; Kinburn, 8.IV.1962, *Acer* sp., J.E.H. Martin (CNC) 3 sex undetermined; 89 mi. N. Pickle Lake, 21.VI.1973, Campbell and Parry (CNC) 1 male; South March, 19.X.1967, A. Smetana (CNC) 1 female; Merivale, 19.VI.1953, deciduous leaf mold (CNC) 1 female; Mer Bleue, 3.VIII.1973, Redner and Starr (CNC) 1 sex undetermined. **Quebec:** Chelsea, 8.VI.1953, E.C. Becker (CNC) 1 sex undetermined; Chelsea, 22.VIII. 1957, J.R. Vockeroth (CNC) 1 sex undetermined; Danford Lake, 30.VI.1953, Berlese deciduous duff, E.C. Becker (CNC) 1 sex undetermined; Rigaud, 15.V.1979, A. Smetana and E.C. Becker (CNC) 1 male, 3 sex undetermined; Rigaud, end Ch. de la Croix, 5.V.1988, A. and Z. Smetana (CNC) 1 female, 1 sex undetermined; 5 km W. Farrellton, Lake Bernard, 8.VII.1973, maple-oak litter, A. Davies (CNC) 2 sex ? undetermined. **Saskatchewan:** Saskatoon, Saskatchewan River, 52.127°N, 106.662°W, 477 m, 6.X.2008, litter sifting, deciduous, B. Godin (BGC) 3 males, 3 females.

UNITED STATES OF AMERICA: **Minnesota:** Ramsey Co., Lake Vadnais, 10.V.1960, soil sample (CNC) 1 male; Brainerd, 10.VI.1965, E.J. Kiteley (CNC) 1 male [new state record].

##### 6. Mocyta sphagnorum

Taxon classificationAnimaliaColeopteraStaphylinidae

Klimaszewski & Webster sp. n.

http://zoobank.org/59167206-821A-42C8-AA6F-13A37A0C2ED6

Figs 8a–h , Map 3

###### Holotype

(female). CANADA, New Brunswick, Restigouche Co., Berry Brook Protected Area, 47.81399°N, 66.75778°W, 26.V.2007, old-growth eastern white cedar swamp, in moss near brook, R.P. Webster (LFC).

###### Paratypes.

CANADA: **Newfoundland:** R.A. Squires Prov. Pk., 23.VII.1970, D.E. Bright (CNC) 1 male. **New Brunswick**, Charlotte Co., Hwy 3 at Deadwater Brook, 45.4745°N, 67.1225°W, 23.IV.2006, Black spruce forest in *Sphagnum*, R.P. Webster (LFC) 1 male, 1 female; Hwy 3 at Deadwater Brook, 45.4745°N, 67.1225°W, 23.IV.2006, Black spruce forest, in sphagnum, R.P. Webster, (RWC) 3 males, 1 female; Carleton Co., Wakefield, Meduxnekeag Valley Nature Preserve, 46.1935°N, 67.8825°W, 19.IV.2005, mixed forest in moist moss, R. Webster (RWC) 1 female; Belleville, Meduxnekeag Valley Nature Preserve, 46.1907°N, 67.6740°W, 4.V.2006, conifer forest area, in moldy conifer duff at base of large white pine, R.P. Webster (RWC) 1 female; “Two Mile Brook Fen”, 46.3619°N, 67.6733°W, 5.VIII.2004, calcareous fen, in sphagnum moss & litter, J. Edsall & R. Webster (RWC) 1 sex undetermined; Restigouche Co., Berry Brook P.N.A., 47.81399°N, 66.75778°W, 26.V.2007, R.P. Webster // Old-growth eastern white cedar swamp, in moss near brook (RWC) 1 male. **Ontario:** 52 mi N Hurkett, Black Sturgeon Lake, 28.VI.1973, R. Parry and J.M. Campbell (LFC) 1 male; Mt. Tremblant Pk., 27.VI.1971, E.J. Kiteley (LFC) 1 female. **Quebec:** Gaspé Co., Mt-Albert, Sommet nord, 1000 m, 18.Vii.1985, sweeping field, F. Génier (LFC) 1 male.

###### Non-types.

Canada, **New Brunswick:** York Co., Canterbury Brown’s Mtn. Fen., 45.8965°N, 67.6344°W, 5.VIII.2004, mixed forest on decaying fungi, J. Edsall and R. Webster (LFC) 1 sex unknown. **Quebec:** Gaspé Co., Mt-Jacques-Cartier, 24.VII.1985, caribou dung, F. Génier and J. Klimaszewski (LFC) 1 male.

###### Etymology.

The specific name *sphagnorum* is an adjective, which derives from the generic name of *Sphagnum*, in the genitive plural, meaning “of the *Sphagnum* plant”, a dominant plant of the habitat where the species was found.

###### Diagnosis.

Body narrowly oval (Fig. 8a), length 2.4–2.7 mm; uniformly brown to almost black, legs and palps yellowish to reddish-brown and antennae uniformly light brown to brown; antennal articles I-IV elongate and V-X variable in length from subquadrate to slightly transverse (Figs 8a, e); pronotum transverse, variable in width, from slightly-to-distinctly broader than elytra [pronotum usually broader in females than in males, Fig. 8e], strongly rounded laterally, and arcuate basally; elytra transverse and slightly shorter than pronotum; abdomen broadly arcuate laterally and with strong macrosetae apically. MALE: median lobe of aedeagus as illustrated with distinct apical structures of median lobe (Fig. 8b); tergite VIII truncate apically (Fig. 8c); sternite VIII slightly produced and rounded apically and with numerous strong macrosetae in apical part of disc, space between base of disc and antecostal suture narrow, antecostal suture arcuate (Fig. 8d). FEMALE: spermatheca pear-shaped with small and shallow apical invagination, stem thin and irregularly coiled posteriorly (Fig. 8f); tergite and sternite VIII truncate apically (Figs 8g, h).

This species may be distinguishable from other *Mocyta* species by its large and dark brown to black pronotum, shape of spermatheca and apical structures of internal sac.

###### Distribution.

This nearctic species is known from Newfoundland, New Brunswick, Quebec and Ontario.

###### Natural history.

In New Brunswick, adults were found in sphagnum moss and litter in calcareous eastern white cedar fens and in a black spruce forest. One individual was collected from moldy conifer duff at the base of a large pine in a mixed forest. Adults were found in April and May in New Brunswick, and June to August elsewhere. This species seems to be associated with moist sphagnum moss.

**Figures 9a–g.**
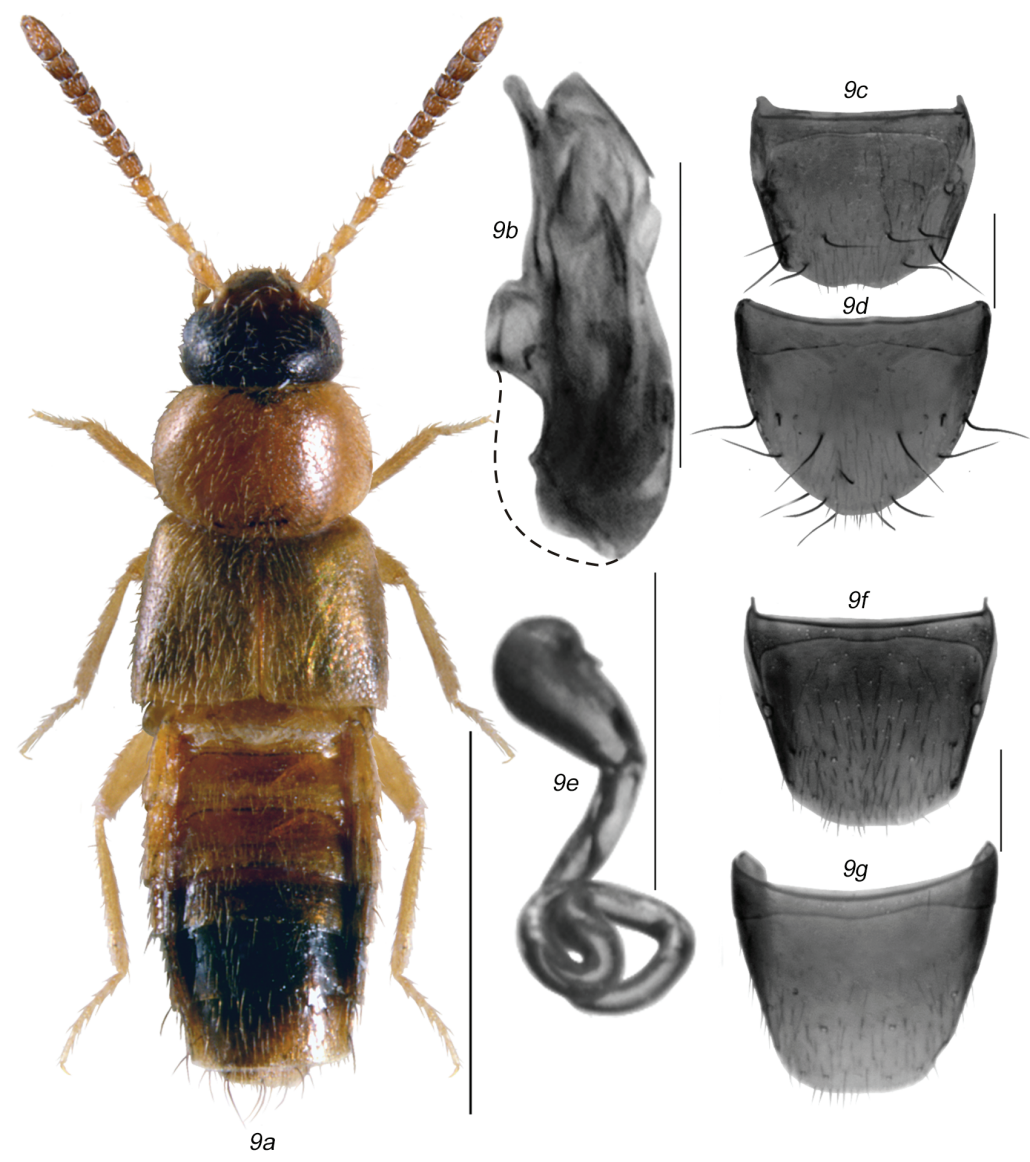
*Mocyta givicollis* (Scheerpeltz) [images based on Types, Austria] (=*Mocyta negligens*): **9a** habitus in dorsal view **9b** median lobe of aedeagus in lateral view **9c** male tergite VIII **9d** male sternite VIII **9e** spermatheca in lateral view **9f** female tergite VIII **9g** female sternite VIII. Broken line indicates original border of bulbus which was distorted during preparations. Scale bar for habitus = 1 mm, and the remaining scale bars = 0.2 mm.

**Figures 10–14.**
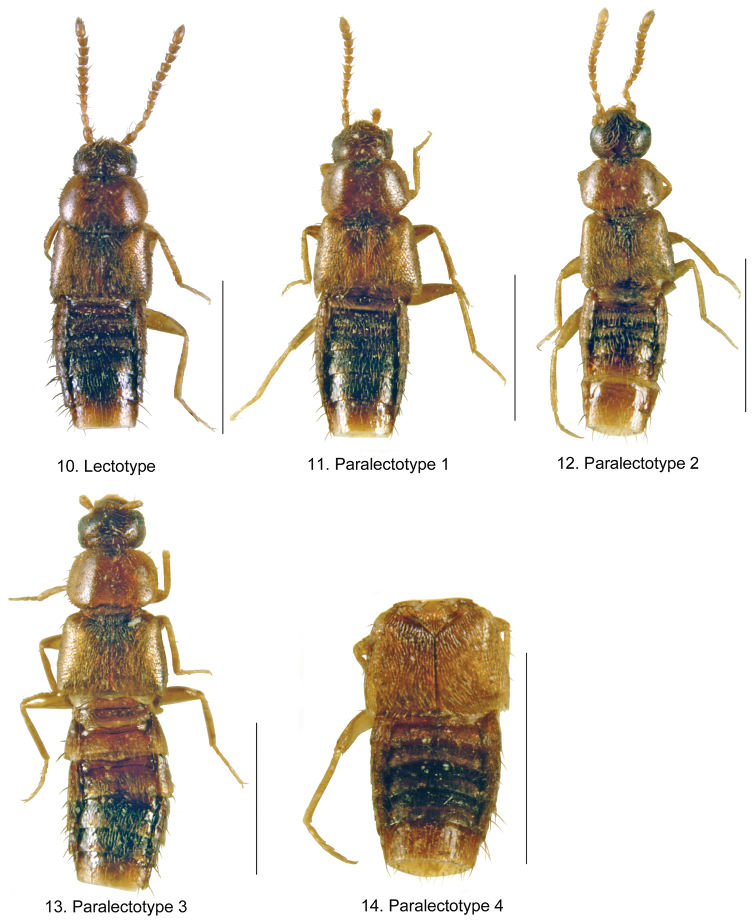
*Mocyta negligens* Mulsant & Rey (=*Mocyta givicollis*) [habitus images in dorsal view, based on types, France]: **10** lectotype **11** paralectotype 1 **12** paralectotype 2 **13** paralectotype 3 **14** paralectotype 4 [abdomen]. Scale bar for habitus = 1 mm.

## Supplementary Material

XML Treatment for Mocyta

XML Treatment for Mocyta amblystegii

XML Treatment for Mocyta breviuscula

XML Treatment for Mocyta fungi

XML Treatment for Mocyta luteola

XML Treatment for Mocyta discreta

XML Treatment for Mocyta sphagnorum
